# Turbidity drives plasticity in the eyes and brains of an African cichlid

**DOI:** 10.1242/jeb.246708

**Published:** 2024-04-08

**Authors:** J. H. Tiarks, Suzanne M. Gray, Lauren J. Chapman

**Affiliations:** ^1^ School of Environment and Natural Resources, The Ohio State University, 2021 Coffey Rd., Columbus, OH 43210, USA; ^2^ Department of Biology, McGill University, 1205 Dr Penfield Ave, Montreal, QC H3A 1B1, Canada

**Keywords:** Sensory system, Ontogeny, Brain size, Optic tectum, Eye size, Morphology

## Abstract

Natural variation in environmental turbidity correlates with variation in the visual sensory system of many fishes, suggesting that turbidity may act as a strong selective agent on visual systems. Since many aquatic systems experience increased turbidity due to anthropogenic perturbations, it is important to understand the degree to which fish can respond to rapid shifts in their visual environment, and whether such responses can occur within the lifetime of an individual. We examined whether developmental exposure to turbidity (clear, <5 NTU; turbid, ∼9 NTU) influenced the size of morphological structures associated with vision in the African blue-lip cichlid *Pseudocrenilabrus multicolor*. Parental fish were collected from two sites (clear swamp, turbid river) in western Uganda. F1 broods from each population were split and reared under clear and turbid rearing treatments until maturity. We measured morphological traits associated with the visual sensory system (eye diameter, pupil diameter, axial length, brain mass, optic tectum volume) over the course of development. Age was significant in explaining variation in visual traits even when standardized for body size, suggesting an ontogenetic shift in the relative size of eyes and brains. When age groups were analyzed separately, young fish reared in turbid water grew larger eyes than fish reared in clear conditions. Population was important in the older age category, with swamp-origin fish having relatively larger eyes and optic lobes relative to river-origin fish. Plastic responses during development may be important for coping with a more variable visual environment associated with anthropogenically induced turbidity.

## Teaser

**Highlighted Article:** The relative sizes of visual features in an African cichlid respond plastically to turbidity during development; for older fish, parental population drives the response of visual traits to turbidity.

## INTRODUCTION

The sensory landscape helps to shape how animals function in their environment by favoring sensory systems that can detect external stimuli against the ambient background. However, human-induced alteration of the sensory landscape can disrupt the relationship between sensory cues and the perception of sensory information. For example, urban noise pollution can mask the calls of songbirds, leading to divergence in songs between urban and rural populations (e.g. great tits, *Parus major*; Slabbekoorn and den Boer-Visser, 2006). In the aquatic environment, eutrophication and increased turbidity (i.e. suspended particles in the water column) can alter the visual scene. This can lead to difficulty choosing an appropriate mate if interspecific nuptial color differences are masked (e.g. *Pundamilia pundamilia* and *Pundamilia nyererei*; Seehausen et al., 2008) or difficulty distinguishing high quality mates (e.g. threespine sticklebacks, *Gasterosteus aculeatus*; Candolin et al., 2016). There is strong evidence for altered behavior in response to rapidly changing sensory landscapes across a wide array of taxa (Candolin and Wong, 2019), with consequences at the interspecific level and within species. We understand less about plasticity in morphological traits that might facilitate behavioural responses and persistence of populations faced with altered sensory landscapes.

Sensory landscapes, sensory organs and sensory processing centers in the brain are directly linked. For visual processes, there is often a match between the ambient light environment and the size and sensitivity of eyes and optic lobes (Dugas and Franssen, 2012; Howland et al., 2004; Huber et al., 1997). Vertebrate eye size tends to follow an allometric relationship with body size (Howland et al., 2004), although there is interspecific variation. In particular, fish show a high degree of variation in eye size among species (Caves et al., 2017; Howland et al., 2004), which may be due to differences among habitats and the complexity of underwater light environments (Sabbah et al., 2010). Caves et al. (2017) found a positive correlation between eye size and visual acuity (i.e. the ability to resolve objects) using a database of 159 ray-finned fishes. This link between eye size and visual acuity among species is likely also found within species where individual differences in visual abilities might be favored depending on the predictability of the visual landscape.

Intraspecific variation in visual abilities has been observed when animals are exposed to different visual landscapes in laboratory rearing studies (e.g. mantis shrimp *Haptosquilla trispinosa*; Cronin et al., 2001). In fish, Kröger et al. (2003) found that blue acara cichlids (*Aequidens pulcher*) showed developmental plasticity in sensitivity to different wavelengths of light. Fish reared under short- to medium-wavelength light were less sensitive to long-wavelength light compared with fish reared under long-wavelength light. This, along with evidence for ontogenetic shifts in visual sensitivity (e.g. Hawryshyn et al., 1989; Nelson et al., 2003; Sabbah et al., 2012), suggests that fish may exhibit developmentally plastic responses in morphological traits associated with visual ability if the visual environment is altered.

Indeed, the size of sensory processing centers (e.g. optic lobes) and the relative size of the brain are often positively correlated and have been linked to ecological functions (e.g. Huber and Rylander, 1992; Huber et al., 1997). Among African Great Lakes cichlids, for example, Huber et al. (1997) found fish from the generally clear Lake Malawi and Lake Tanganyika had relatively more enhanced visual structures than cichlids from the more eutrophic Lake Victoria. Within species, Gonda et al. (2013) found that sticklebacks derived from pond populations with no recent experience with predation risk displayed phenotypic plasticity in olfactory traits (i.e. larger olfactory bulbs when exposed to predation risk), whereas marine populations that are naturally exposed to predation risk displayed no evidence of plasticity. Given the frequent observation of intraspecific variation in brain size and in the size of brain components, it is important to understand whether anthropogenically induced changes in the visual environment can induce an adaptive response in brain size. However, there are likely trade-offs associated with alterations in size given that brains are energetically expensive (Niven and Laughlin, 2008) and demand a large supply of oxygen (Aiello and Wheeler, 1995; Mink et al., 1981). Regardless of the potential benefit of larger brains, brain size is a balancing act between energetic cost and cognitive benefits (Striedter, 2005; Tsuboi et al., 2016).

Turbidity is an environmental stressor that is increasing globally as a result of human-induced alteration of the environment (Dudgeon et al., 2006; Gray, 2016) and can impact the behavior and physiology of aquatic organisms (Killen et al., 2013; Lythgoe, 1980). The direct effects of turbidity can lead to decreased growth and survival (Gray et al., 2016; Reid et al., 2019; Ricciardi and Rasmussen, 1999), and resulting environmental changes from turbidity may also influence a number of visually-guided behaviors including interspecific (e.g. predator–prey relationships; Abrahams and Kattenfeld, 1997; Utne-Palm, 2002) and intraspecific (e.g. reproductive behavior; Candolin et al., 2016; Seehausen et al.,. 2008) interactions. Such behavioral shifts can lead to decreased foraging efficiency (Nieman and Gray, 2020) and potentially disrupt species barriers (Seehausen et al., 2008; van der Sluijs et al., 2011). Reduction in the amount of light and change in spectral composition associated with turbidity could alter investment in vision (e.g. via larger eyes and optic lobes to maintain visual detection thresholds; Dugas and Franssen, 2012; Huber and Rylander, 1992; Huber et al., 1997). In the case of human-induced elevated turbidity due to deforestation, intense agriculture and variable rainfall associated with climate change, a plastic response may be critical to species persistence because of the unpredictable and rapid nature of turbidity fluctuations.

To quantify the direct environmental (plastic) effects of turbidity on visual sensory structures and to test for differences in response between populations exposed to divergent turbidity regimes, we used a full sibling split brood rearing experiment with two populations of a widespread, sexually dimorphic African cichlid *Pseudocrenilabrus multicolor*. This fish is found in diverse habitats, including swamps (clear but tannin-stained; low dissolved oxygen), rivers (high/variable turbidity; high dissolved oxygen; Chapman et al., 2000), and agricultural ditches (high turbidity; high human disturbance; Atkinson and Gray, 2022). We know that morphological (e.g. gills; Chapman, 2021) variation across these populations is at least in part driven by low dissolved oxygen (Atkinson and Gray, 2022; Chapman et al., 2008; Crispo and Chapman, 2010, 2011; McNeil et al., 2016). Male *P. multicolor* also exhibit differences in coloration (McNeil et al., 2016; Atkinson and Gray, 2022); males from swamp populations tend to display more red and darker nuptial coloration, while river populations tend to be more yellow and brighter, potentially matching the ambient backgrounds to enhance detectability (Gray et al., 2012; McNeil et al., 2016). These strong patterns of morphological divergence seem to be maintained by both plastic and genetic effects; however, little is known about potential differences in the visual system of *P. multicolor* among populations that experience different levels of turbidity.

Here, we tested if exposure to low turbidity throughout development elicited a different growth trajectory of sensory structures. Second, we tested if two populations (swamp versus river) responded differently to rearing conditions (clear versus turbid), suggesting a difference in plastic responses between divergent populations. We expected turbidity to favor relatively larger eyes and optic lobes in the brain, though overall brain size may be constrained by dissolved oxygen (see Crispo and Chapman, 2010). Furthermore, we expected to observe a higher degree of plasticity in the response of river-origin fish because the habitat is more variable compared with the largely intact and relatively stable swamp environment.

## MATERIALS AND METHODS

### Field sampling and study populations

Adult *Pseudocrenilabrus multicolor* (Schöller 1903) were captured in 2008 at two sites in the Mpanga River drainage basin of western Uganda and transported to McGill University (Canada) to serve as parental populations for our rearing experiment. Sites chosen represent extremes of turbidity and dissolved oxygen (McNeil et al., 2016; Crispo and Chapman, 2010): Kanyantale [hereafter ‘swamp’; low turbidity, low dissolved oxygen (DO)] and Kamwenge (hereafter ‘river’; variable turbidity, high DO), a site on the Mpanga River downstream of the swamp (see fig. 1 in Crispo and Chapman, 2010). Standard baited minnow traps were set for 2–3 h and checked every 30 min.

### Rearing experiment design

We performed a full sibling split brood rearing experiment to test: (1) whether the development of morphological traits associated with vision varied in response to turbidity and (2) whether responses varied between fish originating from divergent parental populations. Details of the rearing experiment can be found in Gray et al. (2012). Briefly, wild-caught adults from the swamp and river sites were transported live to animal care facilities at McGill University and held under clear, normoxic conditions. Each brood from five sets of known parents, from each of the two focal populations, was divided into two groups post-release from the mother's mouth and randomly assigned to either the clear (mean±s.e.m.=0.81±0.01 NTU over 18 month rearing experiment) or the turbid treatment (mean±s.e.m.= 8.7 NTU±0.10 over 18 month rearing experiment). Therefore, a single family (one male–female pairing with no parents used twice) was split into one clear and one turbid tank for a total of 10 tanks per population. Turbidity was created with bentonite clay kept in suspension using submersible pumps (no filtration) and air stones. Brood size varied from 18 to 48 young (i.e. 9 to 24 fish per tank once the brood was split in two).

Owing to the small tank size (45 liters) broods were culled to 8 fish at ∼6 weeks (between 37 and 45 days post-release; dpr) and to 5 fish at ∼14 weeks (128–142 dpr) around sexual maturity (approximately 4–6 months). The largest male from each tank was used for male–male competition experiments (see Gray et al., 2012) and culled at the end of those trials (287–352 dpr). All remaining fish (i.e. those not culled at earlier dates) were harvested at approximately 18 months (473–567 dpr). We classified all culled fish into two distinct age groups after the rearing experiment based on age at time of harvest (young: 30–200 days, old: 201–600 days) since the size distribution was bimodal (Table 1; Fig. S1). Sex was not considered for young fish because of the difficulty determining sex before sexual maturity; sex was recorded for old fish, but not included in analysis. Young fish varied in average standard length across populations and treatments from 21.2 to 22.8 mm; for older fish, mean standard length ranged from 51.4 to 55.3 mm (Table 1). Fish were euthanized in an overdose of clove oil (1:10 eugenol:ethanol) and preserved in 4% paraformaldehyde.

**Table 1.**
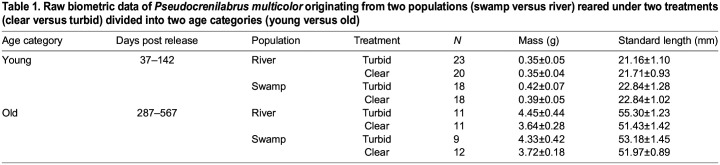
Raw biometric data of *Pseudocrenilabrus multicolor* originating from two populations (swamp versus river) reared under two treatments (clear versus turbid) divided into two age categories (young versus old)

### Morphological measurements

We examined the whole brain and left eye of 65 *P. multicolor* of river origin and 57 of swamp origin (Table 1) to test for variation in sensory morphology across age (young or old), population of origin (swamp or river), and turbidity treatment (clear or turbid). Total body mass (g) and standard length (cm) were measured prior to using standard extraction procedures to remove the brain and eyes (Chapman et al., 2008). All brains and eyes were cleaned to remove excess tissues then stored in 4% paraformaldehyde or 10% buffered formalin.

The brain stem was cut at the posterior margin of the brain for consistent mass measurements across brains (Fig. S2). Total brain wet mass (mg) was measured with an analytical balance (±0.0001 g; Mettler ME104E, Toledo) following Wiens et al., 2014. Each brain was blotted three times with a Kimwipe (Kimberly-Clark, USA) to remove excess preservative, then weighed individually. This procedure was repeated five times over a 5 day period to calculate the mean total brain wet mass (BW).

Digital photographs of each brain and eye were taken using a Nikon SMZ745 T microscope connected to an Infinity 1 camera and NIS Element D software. Brains were imaged in two positions (Fig. S2): dorsal and left lateral side. We estimated the volume (mm^3^) of the optic tectum (OT) using an ellipsoid model (Huber et al., 1997; Pollen et al., 2007; Gonda et al., 2009), where *L* is length, *W* is width and *H* is height (all in mm) with a multiplier of two to account for two optic lobes:
(1)

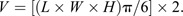


The volume was calculated using OT length (mm) and width (mm) from the dorsal image and height (mm) from the lateral view.

Eyes were photographed in two positions (Fig. S3): dorsal view (with optic nerve down to allow measurement of eye and pupil diameters) and left lateral side (allowing measurement of axial length: front to back of eye). Excess preservative was removed, and then the eye was placed onto a clay mold with a pre-made indent so that the eye laid flat under the microscope. Each measurement of the eye (eye diameter, ED; pupil diameter, PD; axial length, AX) was replicated three times in three different places on the image to obtain a mean value for each eye component.

### Statistical analyses

To test for differences in the relative size of visual traits across age groups, populations, and treatments, we used allometric size standardization, following Hendry and Taylor, 2004):
(2)

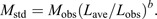


Standardized trait values (*M*_std_) were calculated, where *L*_ave_ is the average size of all the fish used in this analysis (brain mass was used to standardize optic lobe volume and standard length was used to standardize all other morphological traits), *L*_obs_ is the observed standard length (or brain mass) for the individual fish, *M*_obs_ is the observed trait measurement, and *b* is the common within group slope derived from an ANCOVA [log_10_(response variable)]∼population×standard length). All response variables (ED, PD AX, BW, TO) were log transformed to improve normality when determining the common within group slope. Interaction terms that were not statistically significant were removed from the model.

A linear mixed model (LMM) was performed for each brain and eye component separately with population of origin (swamp or river), age group (young: 30–200 dpr, old: 201–600 dpr) and rearing treatment (clear or turbid) as fixed factors and brood (or family) included as a random factor. Brood was removed from the model when not significant, and ANOVAs were performed for that component, respectively. LMMs and/or ANOVAs were then repeated for each age category separately. We tested all assumptions, and the appropriateness of LMM and ANOVA was confirmed. R software was used for all analyses (https://www.R-project.org/), and all LMMs were performed using lme4 (Bates et al., 2015). Using the R package stats, we also conducted a principal component analysis (PCA) using the standardized brain and eye traits to investigate the relationship between visual morphology and variables related to population and rearing. This allowed us to determine if the observed trends were maintained when multicollinearity was removed by PCA. Only factors with Eigen values greater than 1.0 were considered in the analysis. LMMs as described above were repeated on the first two principal components. We used an alpha ≤0.05 to infer a significant difference and alpha ≤0.1 to indicate a trend (Sullivan et al., 2019).

## RESULTS

The final model for brain mass indicated that age group (*F*_1,4,114_=204.6, *P*<0.0001), rearing treatment (*F*_1,4,110_=5.626, *P*=0.0194), and the interaction of age group×rearing treatment (*F*_1,4,110_=3.909, *P*=0.0505) were all significant (or approaching significance at α=0.05), whereas population was not (*F*_1,4,110_=0.0070, *P*=0.9352). Looking specifically at the visual processing center of the brain (OT), age group (*F*_1,4,110_=137.8, *P*<0.0001), population (*F*_1,4,8_=4.652, *P*=0.0627) and the interaction of population×age group (*F*_1,4,110_=8.997, *P*=0.0033), were all significant (or close to with α <0.1) while rearing treatment (*F*_1,4,110_=0.1692, *P*=0.6816) was not. Brood was kept in the BW and OT final models as a random effect (Table 2).

**Table 2.**
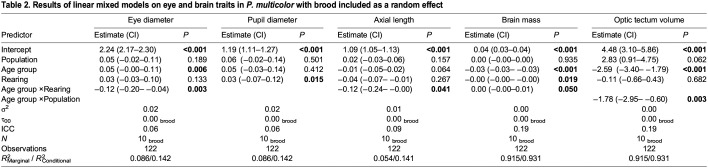
Results of linear mixed models on eye and brain traits in *P. multicolor* with brood included as a random effect

In the final model for eye diameter, age group (*F*_1,4,114_=7.657, *P*=0.0066) and the interaction between age group*rearing treatment (*F*_1,4,110_=9.512, *P*=0.0026) were significant; all other variables were not significant in explaining the observed variation (population, *F*_1,4,8_=2.068, *P*=0.1891; rearing, *F*_1,4,110_=2.286, *P*=0.1334). Similarly, for axial diameter, only the interaction term between age group×rearing treatment (*F*_1,4,110_=4.263, *P*=0.0413) was significant and age group trended toward significance (*F*_1,4,116_=3.490, *P*=0.0644), whereas population (*F*_1,4,7_=2.504, *P*=0.1572) and rearing (*F*_1,4,110_=1.245, *P*=0.2669) were not significant. Rearing (*F*_1,4,112_=6.069, *P*=0.0153) was significant for pupil diameter, but population (*F*_1,4,8_=0.4947, *P*=0.5009) and age group (*F*_1,4,116_=0.6784, *P*=0.4118) were not significant; no interaction terms were significant in explaining the variation in pupil diameter and were subsequently removed from the final model. Brood was kept in the ED, PD and AX final models as a random effect (Table 2).

**Fig. 1.**
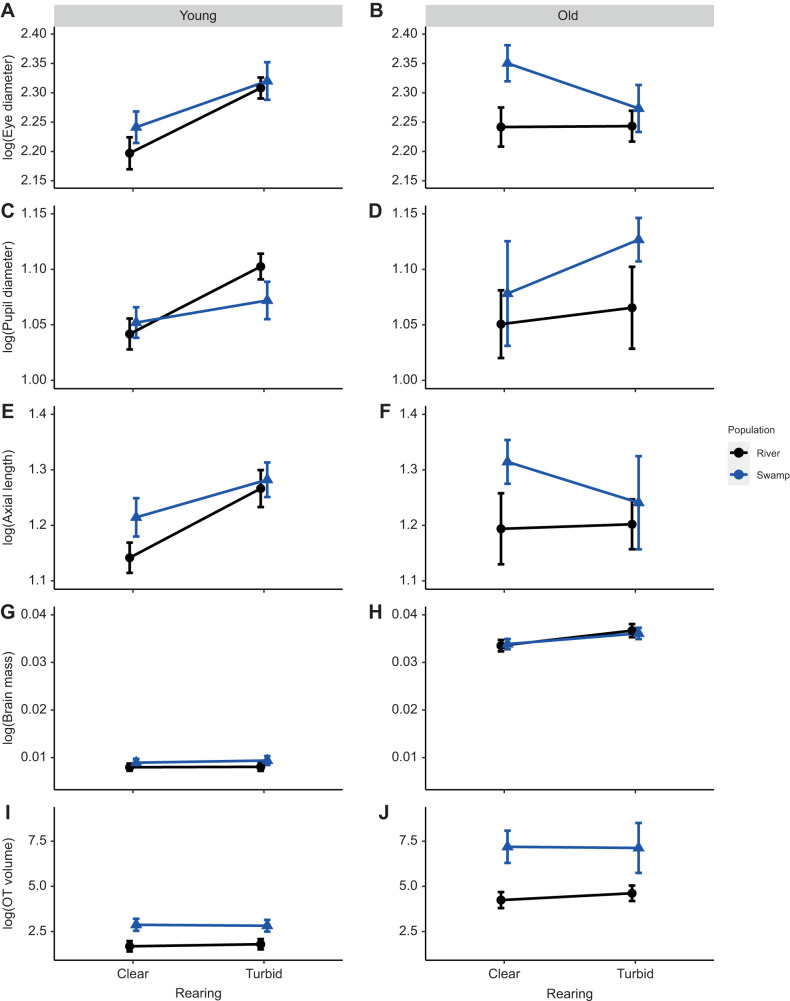
**Morphological measurements across young and old age categories in different rearing treatments for two parent populations of *Pseudocrenilabrus multicolor*.** Log of size standardized eye diameter (ED) (A,B), pupil diameter (PD) (C,D), axial length (AX) (E,F), brain mass (BW) (G,H) and optic tectum volume (OT) (I,J) measurements for different parent populations and rearing treatments.

We repeated the LMMs for each age category separately, given the interaction between rearing×age group for brain mass and population×age group or rearing×age group for eye traits (depending on the visual trait). In young fish (30–200 dpr), rearing treatment had a significant effect on the size of eye traits (ED, PD, AX) in *P. multicolor* but not on brain traits (BW, OT) (Table 3, Fig. 1). During the development of the fish (i.e. young fish), fish reared in turbid water had overall larger eye diameter (*F*_1,76_=14.1361, *P*=0.0003; Fig. 1A), pupil diameter (*F*_1,76_=9.0113, *P*=0.0036; Fig. 1C) and axial length (*F*_1,76_=9.5804, *P*=0.0028; Fig. 1E) compared with fish reared in clear water, but there was no difference in brain mass (*F*_1,70_=0.0726, *P*=0.7883; Fig. 1G) or optic lobe size (*F*_1,68_=0.0004, *P*=0.9835; Fig. 1I). Furthermore, population was not significant in explaining the observed variation in any eye or brain traits of fish in the young category. Brood was kept in the model if it was significant; otherwise, it was removed and an ANOVA was run. All other interaction terms were not significant and removed from the model.

**Table 3.**
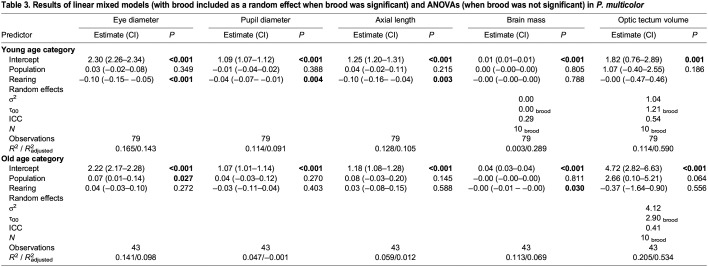
Results of linear mixed models (with brood included as a random effect when brood was significant) and ANOVAs (when brood was not significant) in *P. multicolor*

Analysis of the old age category (201–600 dpr; Table 3) revealed that population had a significant effect on relative eye diameter (*F*_1,33_=5.3122, *P*=0.0265; Fig. 1B) and trended toward a significance effect on optic tectum size (*F*_1,33_=4.4410, *P*=0.0641; Fig. 1J), while rearing treatment only had a significant effect on brain mass (*F*_1,40_=5.0474, *P*=0.0303; Fig. 1H). Older swamp fish had relatively larger eye diameters and optic tecta compared with river fish, whereas fish reared in turbid water had larger brains than those reared in clear water, regardless of population of origin.

**Fig. 2.**
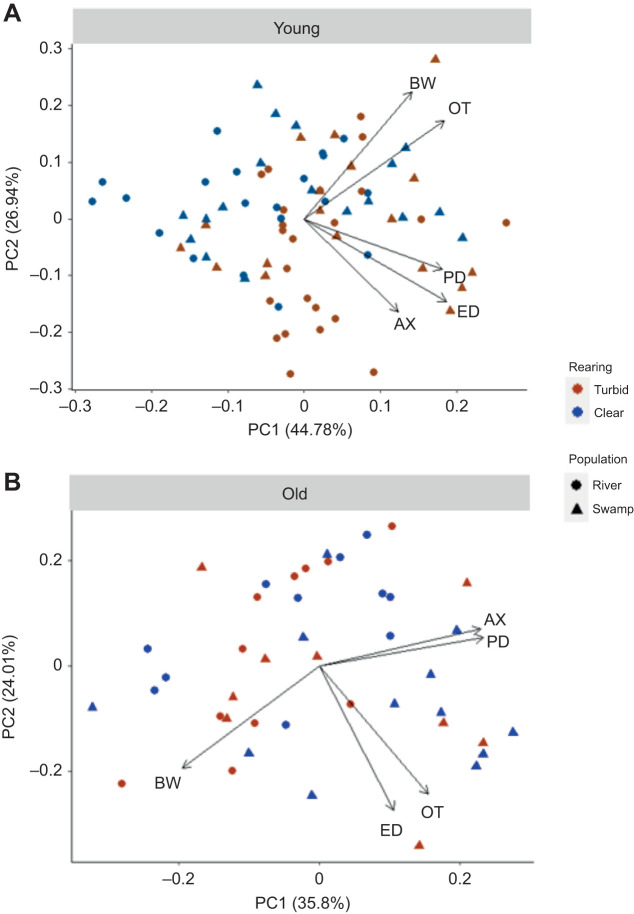
**Ordination diagram from first two principal components on eye and brain traits for *P.*** ***multicolor*****.** River population (brown symbols), swamp population (blue symbols), turbid rearing (circles), clear rearing (triangles) for the young age category (A) and the old age category (B). All metrics were size standardized prior to analysis allowing for comparison of relative values as indicated in the Materials and Methods.

Principal component analysis (PCA) was performed separately on the standardized eye and brain traits for the young and old age categories. The analysis for the young age category yielded two axes with Eigen values >1, with the first component (PC1) explaining 44.78% and the second component (PC2) explaining 26.94% of the total variance (cumulatively explaining 71.72% of the total variance) (Tables S1 and S2). Although all brain and eye metrics positively associated with the PC1 axis for the young age category, PC2 revealed a divergence in eye traits (negatively associated to the axis) and brain traits (positively associated to the axis) (Fig. 2A; Tables S1 and S2). The analysis for the old age category also yielded two axes with Eigen values >1, with the first component (PC1) explaining 35.80% and the second component (PC2) explaining 24.01% of the total variance (cumulatively explaining 58.81% of the total variance). For the old age category, most traits were positively associated with the PC1 axis, with the exception of brain mass. The PC2 axis showed OT, BW, and ED positively associated and PD and AX negatively associated (Fig. 2B; Tables S1 and S2).

We ran LMMs on the two principal components separately for the two age groups. The population×rearing interaction term was removed since it was not significant in either model, and brood was kept as a random effect since it was significant in both models. In the young category, rearing treatment had a significant effect on both PC1 (*F*_1,68_=10.117, *P*=0.0022) and PC2 (*F*_1,69_=9.441, *P*=0.0030); however, no population effect was found during development on either PC1 (*F*_1,7_=1.390, *P*=0.2758) or PC2 (*F*_1,8_=0.0901, *P*=0.7709) (Table 4). Conversely, for the old age category there was no significant effect of rearing treatment on either PC1 (*F*_1,33_=0.4936, *P*=0.4872) or PC2 (*F*_1,40_=0.3710, *P*=0.5459); however, the effect of population was significant for PC1 (*F*_2,40_=5.6144, *P*=0.0190) and trended toward significance for PC2 (*F*_2,40_=2.977 *P*=0.124) (Table 4).

**Table 4.**
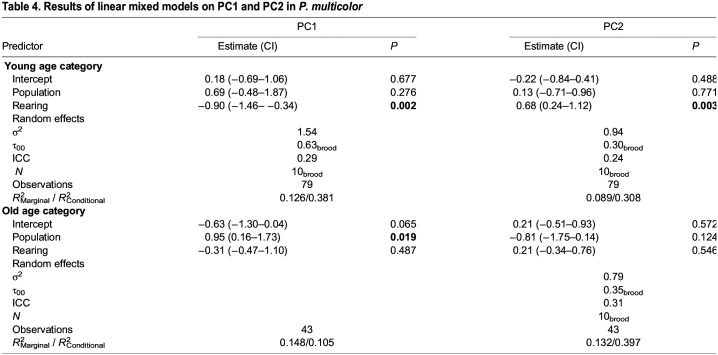
Results of linear mixed models on PC1 and PC2 in *P. multicolor*

## DISCUSSION

Our results reveal that the age of a fish drives the morphological response of eye and brain traits associated with vision, such that the size-standardized visual systems of young fish and old fish responded differently to turbidity in our reciprocal common garden experiment. We found an overall difference in the pattern of eye and brain sizes relative to body size between young and old fish. Older fish had larger brains relative to their length and larger optic lobes relative to brain mass compared to that of younger fish. Relative eye size did not change with age; however, younger fish reared in turbid water had relatively larger eye structures (ED, AX, PD) than fish reared in clear conditions, regardless of population of origin. We discuss these patterns below.

Although the brains of teleost fish continue to grow throughout their lifetime (Brandstätter and Kotrschal, 1990; Ekström et al., 2001; Leyhausen et al., 1987), the relative difference we observed between age categories could be a result of different visual (or other sensory) demands at different life stages; these relative changes may reflect changes in brain morphology triggered by sexual maturation. For example, Kotrschal et al. (2014) found that early maturing Atlantic salmon (*Salmo salar*) had relatively larger brains than slower growing fish, suggesting that more energy may be put into developing processing centers in the brain in fish requiring the cognition necessary for courtship and mating. Many cichlids go through ontogenetic shifts in visual sensitivity (Carleton, 2009; Gray, 2021) where they switch the expression of their cone opsin genes (short wavelength to longer wavelength) to facilitate color vision when they transition from juveniles to adults (Carleton et al., 2016). Additional ontogenetic changes, such as microhabitat use, may affect the photic environment experienced during different life stages, and subsequently, visual morphology. We currently do not know if *P. multicolor* shifts microhabitats or prey items during ontogeny; however, we do catch a large size range of fish in the same minnow traps and seine hauls (S.M.G., personal observation), suggesting they are found in similar locations with similar photic environments. Since *P. multicolor* is sexually dimorphic, color vision may be particularly important for mature fish, and additional neural processing for that sensory information may be necessary (e.g. larger brains). Since we only assessed gross morphology via volume of the optic lobes, it is possible that correlated changes may not be observed in anatomical size differences. A more thorough examination of the internal structures (e.g. subcellular and laminated compartments) may be needed to pinpoint a mechanistic explanation and observation of optic tecta plasticity.

Rearing treatment had a significant effect on the size of visual sensory structures in the young age group, indicating a plastic response in eye morphology under relatively low levels of turbidity. Larger eyes are associated with higher visual acuity at an interspecific level (Caves et al., 2017). Within species, phenotypic plasticity is one mechanism that could allow fish to respond to and persist in a changing visual environment (West-Eberhard, 2003). The plastic response of eye traits to turbidity during 30–200 days post release was found in both the river and swamp populations, suggesting that plasticity in response to a visual stressor is independent of the origin of the fish. Even though plasticity can be costly to maintain, phenotypic plasticity may be crucial for an organism to respond and cope with a rapidly changing landscape. However, the extent to which phenotypic plasticity will contribute to an adaptive advantage in fluctuating environments depends on the rate and duration of environmental change (Fox et al., 2019; Merilä and Hendry, 2014; Van Baaren and Candolin, 2018). Since water turbidity can shift rapidly (e.g. in response to rainfall with associated runoff or more slowly in the context of seasonal warming and algae accumulation), it would be useful to explore plasticity in response to different exposure times within age groups. This may be especially important even after development in highly degraded systems where future conditions are less predictable (Merilä and Hendry, 2014).

Our results suggest that exposure to elevated turbidity promotes the growth of larger eyes during development, which could be tied to ontogenetic shifts and the need to maintain visual acuity under altered lighting conditions; however, there was not a concomitant enlargement of the brain size or optic lobe size in young fish. The factors favoring the evolution of plasticity in vision (and other sensory modalities) are complex given that both the sensing traits (e.g. eye morphology) and processing centers (e.g. brain morphology) need to be aligned for the correct interpretation of sensory cues (DeWitt et al., 1998; Wiens et al., 2014). In a study that looked at the coordinated evolution of brain and eye morphology in Trinidadian killifish (*Anablepsoides hartii*), there was no correlation between overall brain size and eye size (Howell et al., 2021), similar to our results; however, the same study showed a correlation between components of the brain (cerebellum, optic tecta and telencephalon) and eye size. Interestingly, our results suggest that eye and brain morphology may not respond in concert to alterations of the visual environment: eye and brain morphology responded independently to changes in the visual landscape at different stages of development. Further investigation into the internal structures of the optic tectum and to other components in the brain could provide additional insight into how brain morphology is affected by low levels of turbidity. Specifically, future studies in *P. multicolor* should investigate the telencephalon, as it directly receives information from the retina and is linked to behaviors such as mating (Cooper et al., 1989; Luiten, 1981).

Interestingly, population, rather than rearing environment, explained some variation in older fish (Table 3). Whereas the brain itself was larger due to the rearing effect of elevated turbidity in older fish, the size of the optic lobes relative to the size of the brain within the old age category was driven by differences between populations. Earlier studies on *P. multicolor* have documented a high level of plasticity in brain size when fish were reared under high-oxygen versus low-oxygen conditions, with smaller brains in hypoxia-reared fish (Crispo and Chapman, 2010; Wiens et al., 2014). In these studies, traits were measured only on adult fish, so we do not know if the plasticity reflected age-specific effects. Surprisingly, swamp fish had larger optic lobes compared with those in river fish, regardless of rearing treatment. This may relate to the complexity of their natural habitat. Dobberfuhl and colleagues explored the relationship between visual acuity and habitat complexity in three species of African cichlid fishes [*Xenotilapia* (formerly: *Asprotilapia*) *leptura*, *Xenotilapia spilotera* and *Xenotilapia flavipinnis*] (Dobberfuhl et al., 2005; Shumway, 2010). They found that the species from more complex habitats had a greater ability to resolve spatial information. The trend is similar for eye morphology, such that there is a correlation between more complex habitats, higher visual acuity and larger eye size relative to body size (Caves et al., 2017). Caves et al. (2017) also found that visual acuity was higher in bright clear habitats and there was a relatively lower investment in eyes for species that live in darker habitats. While our study did not directly quantify habitat complexity, the swamp (clear) habitat of the parental fish used in our study is largely intact and densely vegetated with papyrus (*Cyperus papyrus*), providing a buffer against environmental fluctuations (except in extreme circumstances such as flooding). Conversely, the river (turbid) habitat consists of relatively open water with sparse aquatic vegetation and is subject to variable turbidity levels as a result of human-induced environmental change (e.g. deforestation and agriculture, intensified and variable rainfall with climate change). It is possible that the clear water and complex structure that characterizes the swamp environment may contribute to greater visual acuity that could be associated with larger optic lobes.

### Conclusion

Plastic responses in sensory traits associated with changes in the visual landscape may allow some populations/species to cope and persist in the face of human-induced environmental change. Globally, the visual landscape of freshwaters is changing and compensatory visual mechanisms are therefore expected in aquatic environments altered by humans for species that rely on vision to survive. Our findings support developmental plasticity in morphological traits associated with the detection and processing of visual stimuli in response to elevated turbidity during rearing. We also demonstrate that young fish reared in turbid waters grew relatively larger eyes, regardless of their population of origin; however, we show that population of origin was significant in explaining variation eye and brain morphology in the older age category. These trends were maintained when multicollinearity was removed, with rearing explaining variation in the PC1 and PC2 axis for the young age category and population explaining PC1 variation in the old age category (but not PC2 variation). Collectively, this research provides a better understanding of the effect of turbidity on African cichlid visual sensory systems and contributes to the growing knowledge of how animals respond differently to environmental change.

## Supplementary Material

10.1242/jexbio.246708_sup1 Supplementary information
