# Supplementary material for: Turbidity drives plasticity in the eyes and brains of an African cichlid
Source: J Exp Biol. 2024 Apr 8;227(7):jeb246708. doi: 10.1242/jeb.246708 (PMC11058630; doi:10.1242/jeb.246708)
Supplement: Supplementary information [file jexbio-227-246708-s1.pdf]

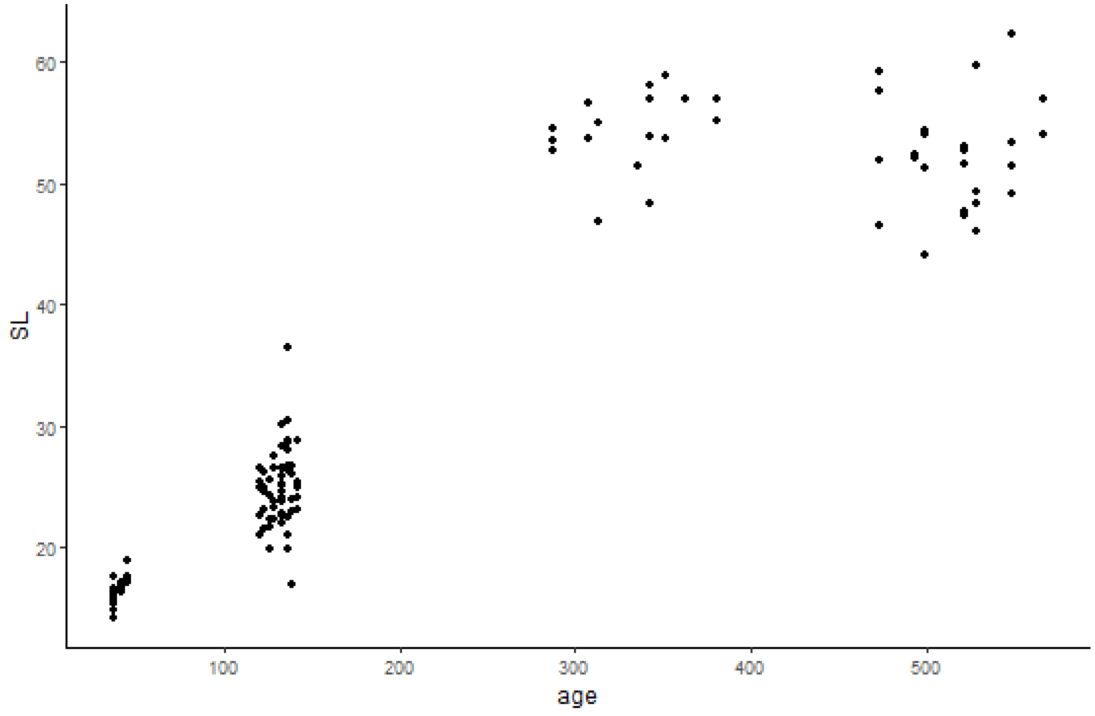

Fig. S1. Plot showing age (days) and standard length (cm) showing distinct age categories.

Journal of Experimental Biology • Supplementary information

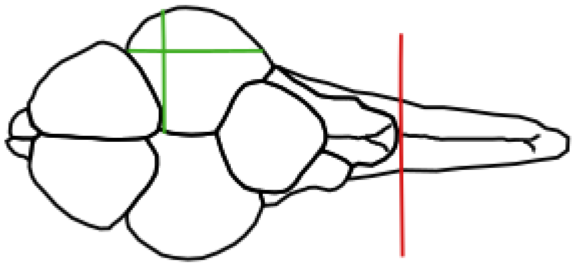

(A)

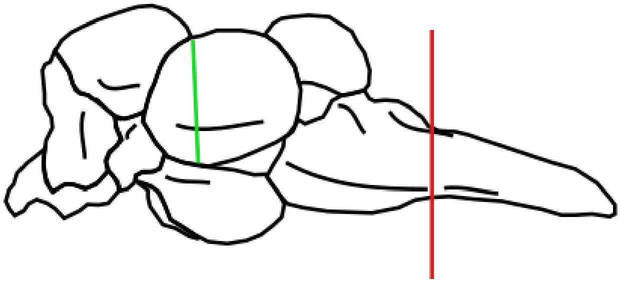

(B)

Fig. S2. (a) Graphical representation of dorsal view of *P. multicolor* brain demonstrating measurement of length and width of optic tectum (green) and posterior margin where brain stem was cut (red). (b) Graphical representation of lateral view of *P. multicolor* brain demonstrating measurement of lateral height of optic tectum (green) and posterior margin where brain stem was cut (red).

Journal of Experimental Biology • Supplementary information

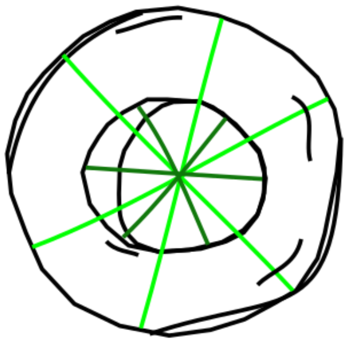

(A)

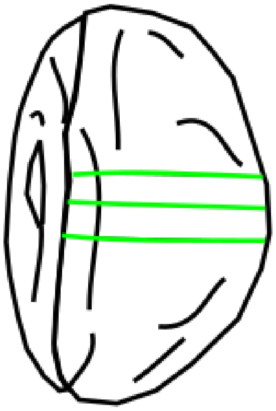

(B)

Fig. S3. (a) Graphical representation of dorsal view of *P. multicolor* eye demonstrating measurement of eye diameter (green) and pupil diameter (dark green). (b) Graphical representation of lateral view of *P. multicolor* eye demonstrating measurement of axial length (green).

Journal of Experimental Biology • Supplementary information

Table S1. Factor loads from principal component analysis separated by age category.

(A) Young age category

	PC1	PC2	PC3	PC4	PC5
Optic tecta	0.4971817	0.4678355	-0.03895129	-0.1679197	0.71008874
Brain mass	0.3824360	0.6085368	0.27974970	0.1170856	-0.62566504
Eye diameter	0.5048947	-0.3945325	-0.13591874	-0.7064542	-0.26809316
Pupil diameter	0.4903607	-0.2407657	-0.56015005	0.6189008	-0.06907951
Axial length	0.3334377	-0.4440595	0.76679914	0.2756414	0.16634696

(B) Old age category

	PC1	PC2	PC3	PC4	PC5
Optic tecta	0.3636844	-0.5736010	0.5054719	-0.04117115	0.53058331
Brain mass	-0.4570584	-0.4582524	0.3313396	0.51133325	-0.45809905
Eye diameter	0.2506178	-0.6454860	-0.6114977	-0.23147162	-0.30500821
Pupil diameter	0.5501477	0.1277193	-0.2294590	0.79163901	0.04100685
Axial length	0.5416267	0.1673983	0.4562142	-0.23784830	-0.64336230

Journal of Experimental Biology • Supplementary information

Table S2. Principal components contribution scores to the observed variance for the principal component analysis separated by age category.

(A) Young age group

	PC1	PC2	PC3	PC4	PC5
Standard deviation	1.4963	1.1606	0.8744	0.60968	0.52714
Proportion of variance	0.4478	0.2694	0.1529	0.07434	0.05558
Cumulative proportion	0.4478	0.7172	0.8701	0.94442	1.00000

(B) Old age group

	PC1	PC2	PC3	PC4	PC5
Standard deviation	1.338	1.0956	0.8958	0.7943	0.7591
Proportion of variance	0.358	0.2401	0.1605	0.1262	0.1152
Cumulative proportion	0.358	0.5981	0.7586	0.8848	1.0000
